# An Analysis of Skin of Color Content on TikTok

**DOI:** 10.2196/33340

**Published:** 2022-03-01

**Authors:** Kayd J Pulsipher, Anthony Concilla, Colby L Presley, Melissa R Laughter, Jaclyn Anderson, Emily Chea, Kristina Lim, Chandler W Rundle, Mindy D Szeto, Robert Dellavalle

**Affiliations:** 1 College of Osteopathic Medicine Rocky Vista University Ivins, UT United States; 2 Philadelphia College of Osteopathic Medicine Philadelphia, PA United States; 3 Division of Dermatology Lehigh Valley Health Network Allentown, PA United States; 4 Department of Medicine University of Texas at Austin Dell Medical School Austin, TX United States; 5 Department of Pathology School of Medicine Stanford University Stanford, CA United States; 6 Department of Dermatology Duke University School of Medicine Durham, NC United States; 7 Department of Dermatology University of Colorado Anschutz Medical Campus Aurora, CO United States; 8 Dermatology Service Rocky Mountain Regional Medical Center US Department of Veteran Affairs Aurora, CO United States; 9 Department of Epidemiology Colorado School of Public Health University of Colorado Anschutz Medical Campus, Aurora, CO United States

**Keywords:** internet, social media, TikTok, skin of color, SoC, influencer, user engagement, hashtag, dermatologist

The US population has continually diversified in the past decade (2010-2019), with recent data estimating 40% of US citizens identify with a race or ethnic group other than White [1]. Social media is an impactful outlet for dissemination of dermatologic education. Most recently, TikTok has emerged as a leading social media platform, reaching over 1 billion users daily. Previous studies indicate a growing presence of dermatologists on TikTok, while also highlighting the need for increased involvement to combat the spread of misinformation [2]. Wells et al [3] previously evaluated skin of color (SoC) posts on the social media platform Instagram, with findings identifying that dermatologists are underrepresented among those producing SoC posts. Considering the exponential growth of TikTok, we aimed to perform a similar study evaluating the credentials of “influencers” who produce SoC dermatologic posts on TikTok.

Data were collected from TikTok in March 2021. General dermatology and SoC dermatology posts were identified by searching individual hashtags (Table 1). A list of SoC-specific terms was generated using common SoC pathologies from the Skin of Color Society website [4]. The top 10 posts associated with each hashtag, as determined by the TikTok algorithm, were analyzed. Posts not relevant to dermatology were excluded.

The user profile of each post was analyzed to classify the creator. Posts were also classified as advertisements, educational, or promotional. Posts were classified as advertisements if the post attempted to sell a specific dermatological product or service. Posts that provided educational information to the viewer without advertising were classified as educational. Posts were classified as promotional if they were self-promoting of the TikTok user/poster. User engagement (number of likes, comments, shares, and views) was also recorded for each post.

Dermatologists were responsible for 20% (32/160) of the SoC posts on TikTok, while influencers produced 36% (57/160) of SoC posts. Patients and physicians other than dermatologists each produced 14% (23/160) of the SoC posts, while hairstylists, estheticians, medical students, and naturopathic doctors produced 8% (13/160), 6% (10/160), 2% (3/160), and 2% (3/160) of SoC posts, respectively. Of the 16 SoC hashtags analyzed, only one (#skinofcolor) had dermatologists producing the majority of the posts. Patients, influencers, and hairstylists produced the highest percentage of the top posts for all other SoC hashtags. The hashtag #acne garnered the highest user engagement but the related posts were primarily personal and noneducational (Table 1).

**Table 1:** Skin of color (SoC) hashtag search terms and their top 10 posts’ average user engagement, post type, and creator type on TikTok.

Hashtag	Average likes (IQR)	Average comments (IQR)	Average shares (IQR)	Average views (IQR)	Types of top 10 posts (E/P/A)^a^	Most common creator type (number of top 10 posts produced)
#skinofcolor	4416 (1192)	111 (56)	94 (103.0)	71,459 (83,000)	7/1/2	Dermatologist (4/10)
#acne	3,290,000 (1,200,000)	30,567 (26,475)	113,160 (97,350)	24,690,000 (14,525,000)	2/5/3	Patient (7/10)
#postinflammatoryhyperpigmentation	1336 (914)	33 (26)	58 (17)	18,458 (12,845)	5/1/4	Influencer (7/10)
#PIH	15,194 (23,270)	281 (269)	186 (155)	160,130 (273,700)	4/1/5	Influencer (5/10)
#razorbumps	28,017 (15,275)	114 (103)	1032 (1238)	272,879 (157,125)	3/0/7	Influencer (5/10)
#melasma	89,470 (84,125)	2101 (516)	4997 (2923)	1,312,210 (781,450)	5/4/1	Influencer (3/10)
#keloid	106,240 (68,150)	1752 (1097)	2961 (2405)	1,089,230 (1,211,900)	5/5/0	Patient (7/10)
#tractionalopecia	2475 (2284)	67 (42)	161 (119)	32,507 (42,331)	3/4/2	Patient (7/10)
#eczema	149,120 (65,925)	1475 (893)	3675 (4564)	1,986,730 (1,410,100)	4/5/1	Patient (6/10)
#vitiligo	898,640 (520,700)	8286 (6504)	4637 (5994)	4,940,000 (3,350,000)	0/10/0	Patient (8/10)
#melanoma	51,400 (47,000)	545 (848)	1181 (1646)	564,960 (441,450)	4/6/0	Patient (4/10)
#psoriasis	97,520 (57,200)	2448 (2081)	1380 (1518)	859,840 (365,450)	1/7/2	Patient (8/10)
#sarcoidosis	3030 (1226)	109 (52)	66 (21)	78,842 (34,975)	4/6/0	Patient (8/10)
#seborrheicdermatitis	19,533 (7610)	294 (190)	591 (212)	299,320 (169,475)	5/5/0	Patient (6/10)
#dandruff	575,190 (468,925)	5388 (8669)	6670 (10,976)	3,558,520 (4,075,000)	4/4/2	Hairstylist (6/10)
#hairbreakage	44,7430 (32,601)	379 (353)	2929 (144)	592,610 (421,450)	6/3/1	Influencer (5/10)

^a^E/P/A: educational, promotional, advertisement.

Social media has been described as the new horizon for dermatological education [5]. However, our analysis reveals dermatologists have a small contribution (20%) to SoC posts on TikTok. This finding suggests patients with SoC using TikTok are obtaining dermatologic information from an alarming number of posts by socially recognized “influencers” who lack professional credentials, such as licensing or board certification, as a qualified medical doctor or clinician. Due to socioeconomic, cultural, and various other factors, patients with SoC in the United States have lower rates of in-person health service utilization when compared to White individuals [6].With the plethora of dermatologic information available on TikTok, lower rates of health service utilization may be perpetuated as patients with SoC use online resources for dermatologic care. Quality control is a major challenge associated with social media, which enables the circulation of inaccurate information. TikTok, however, offers a “duet” feature, which grants dermatologists the option to post public replies to and corrections of inaccurate videos. This feature is commonly used by dermatologists and other health care professionals on TikTok to reinforce professional medical advice and limit the spread of misinformation [2]. Limitations of our study include classifying creators based on TikTok profile descriptions without license/certification verification. Our study provides a mere snapshot of top creators for SoC dermatologic care due to the continually evolving nature of TikTok. Our study suggests TikTok is an important social media platform that dermatologists should consider using for educating and promoting correct dermatologic practice for patients with SoC.

## Abbreviations

SoC: skin of color

## References

[1] The nation is diversifying even faster than predicted. Brookings.

[2] Presley CL, Pulsipher KJ, Rietcheck HR, Szeto MD, Laughter MR, Dellavalle RP (2022). Reply to "Dermatologists in social media: A study on top influencers, posts, and user engagement": Dermatologist influencers on TikTok. J Am Acad Dermatol.

[3] Wells TM, Rundle CW, Szeto MD, Presley C, Dellavalle RP (2020). An Analysis of Skin of Color Dermatology Related Content on Instagram. J Drugs Dermatol.

[4] Skin of Color Society.

[5] Amir M, Sampson BP, Endly D, Tamai JM, Henley J, Brewer AC, Dunn JH, Dunnick CA, Dellavalle RP (2014). Social networking sites: emerging and essential tools for communication in dermatology. JAMA Dermatol.

[6] Manuel JI (2018). Racial/Ethnic and Gender Disparities in Health Care Use and Access. Health Serv Res.

